# Non-invasive contactless analysis of an early drawing by Raffaello Sanzio by means of optical methods

**DOI:** 10.1038/s41598-022-18600-2

**Published:** 2022-09-16

**Authors:** Diego Quintero Balbas, Alice Dal Fovo, Letizia Montalbano, Raffaella Fontana, Jana Striova

**Affiliations:** 1 National Research Council—National Institute of Optics (CNR-INO), L.go E. Fermi 6, 50125 Florence, Italy; 2 Laboratorio di Restauro Cartacei e Membranacei, Opificio delle Pietre Dure-MiC, Viale F. Strozzi 1, 50129 Florence, Italy

**Keywords:** Imaging studies, Raman spectroscopy, Scientific data, Applied optics, Optical techniques

## Abstract

Studying highly valuable and fragile Renaissance drawings requires a non-invasive contactless analytical approach. In this work, we study an early drawing by Raffaello Sanzio, one of the most important artists of the Italian Renaissance, realized during his stay in Florence (1504–1508). Our analyses aimed to reveal the features of the paper support significant for its dating, identify and map the drawing media to understand the artist’s technical approach, and document the drawing condition with imaging and single-sited optical methods. Reflectance Vis–NIR multispectral imaging spectroscopy elaborated with False-Colour and Principal Component Analysis provided information about the paper support and the drawing media. Laser scanning micro-profilometry and Optical Coherence Tomography allowed revealing the superficial micro-scale features of the support. The chemical composition of the different drawing materials was characterized by μ-Raman spectroscopy, which provided also some evidence of the conservation history of the drawing. Integration of spectroscopic and imaging data shows that Raffaello used different dry drawing media (carbon-based and Pb stylus) to sketch the figure and then refined the details and shadows with iron-gall ink. The paper presents a methodological approach for the analytical examination of fragile paper artworks.

## Introduction

Renaissance drawings—originally conceived as a preparatory phase (e.g., sketches and cartoons) during artwork production—are highly valuable because of their quality and their documentary relevance for studying artistic processes and influences^1,2^. For this reason, in the last few years, scholars have focused on describing and comparing the technical approaches of different artists from the same period^3–11^. Drawings are apparently simple objects, being constituted by only two main components, namely the support (e.g., paper or parchment) and a relative limited variety of drawing media (e.g., metal points, inks, and carbon-based media). However, the fragile nature and the generally reduced dimension of paper and membranous artefacts require a non-invasive multi-analytical approach combining complementary imaging and spectroscopic methods, in order to obtain a comprehensive knowledge of the object without endangering its structural integrity^11^.

Combined optical analysis allows for a detailed examination of the artworks, which turns valuable either to monitor their condition and/or record fainted details not visible to the naked eye^7,12^. The documentation provided by the optical methods reduces the manipulation of the object itself, thus contributing to its preservation. Imaging analysis provides effective visualization tools for the dissemination of the results to different audiences^13^.

Regarding the support, additionally to the direct observation under different light sources and with magnification tools, paper-based artefacts can be studied using beta- and low-intensity X-ray radiography^14–16^. Most recently, laser micro-profilometry and optical coherence tomography (OCT) have shown promising results by registering superficial features at a microscopic level^7^. To date, the morphological features of Renaissance drawings, primarily useful for their dating (i.e., providing information on technical details to the production period, such as laid lines dimensions and distribution), are poorly documented in literature.

Contrariwise, there is a consistent number of publications on drawing media. Analyses have been performed using imaging methods, such as Infrared Reflectography (IRR)^10,17^, UV-reflectance and induced fluorescence imaging^10^, and visible and near-infrared multi- and hyper-spectral imaging^18^. Single-sited and mapping methods, such as X-ray fluorescence spectroscopy and imaging (XRF and MA-XRF)^8,10,11,19,20^, Fibre Optics Reflectance Spectroscopy (FORS)^8,21^, Fourier Transform Infrared (FTIR) and Raman spectroscopies^9,11,22^, as well as particle-induced X-ray emission (PIXE)^17,23,24^, and synchrotron-based techniques^25^ provide chemical characterization of the materials that profitably integrates with imaging data. The application of multivariate analysis on imaging^7^ or spectroscopic data aids to highlight and discriminate close-related materials, such as carbon-based black media^26^. Integral information on the drawings’ materials and conservation state and history can only be achieved by a multianalytical approach, such as the combination of elemental (e.g., XRF), molecular (e.g., FTIR and/or Raman) and reflectance spectroscopies and imaging and topographic measurements, (e.g., Atomic Forse Microscopy, OCT)^7,20^.

In this work, we report on the analyses on a drawing by Raffaello Sanzio, the *Cristo Morto* (Inv. 84), which represents the most important item in the collection of the Marucelliana Library (Florence, Italy). The drawing was exhibited in January 2022 in a dedicated exhibition “…Per fare notomia. Il Cristo anatomico di Raffaello nella Biblioteca Marucelliana di Firenze” at the Marucelliana Library in Florence, curated by Silvia Castelli and Piera Giovanna Tordella, where important art historical and scientific results were presented^27^. The full RGB image of the drawing can be consulted in several resources^28,29^. The drawing was realized during Raffaello’s stay in Florence (1504–1508), where he studied the work of Leonardo and Michelangelo. It became part of the Marucelliana Library collection thanks to the Francesco di Ruperto Marucelli bequest in 1738, and originally was part of the Book C entitled *Disegni Istoriati*^28^*.*

We exploited a set of optical methods, namely multispectral reflectance imaging spectroscopy (RIS) in the visible (Vis) and near-infrared (NIR) regions, post processed with False-Colour (FC) and Principal Component Analysis (PCA), to study the paper support, explore the homogeneity of the drawing media, and understand the artist’s working process. We analysed the micromorphology of the paper sheet with laser scanning micro-profilometry and Optical Coherence Tomography (OCT), and we used micro-Raman spectroscopy (μ-Raman) to chemically characterize the drawing media and reconstruct the conservative history of the drawing. Data were compared to reference drawing materials, which were widely used in the Renaissance period. The results allowed the identification of the different drawing materials used by the artist, as well as an understanding of his technical approach: the use of dry media for sketching and ink for completing and detailing the image. The data also provided insight into the conservation condition of the drawing, including previous undocumented treatments.

## Results and discussion

### Paper support analysis

**Figure 1:**
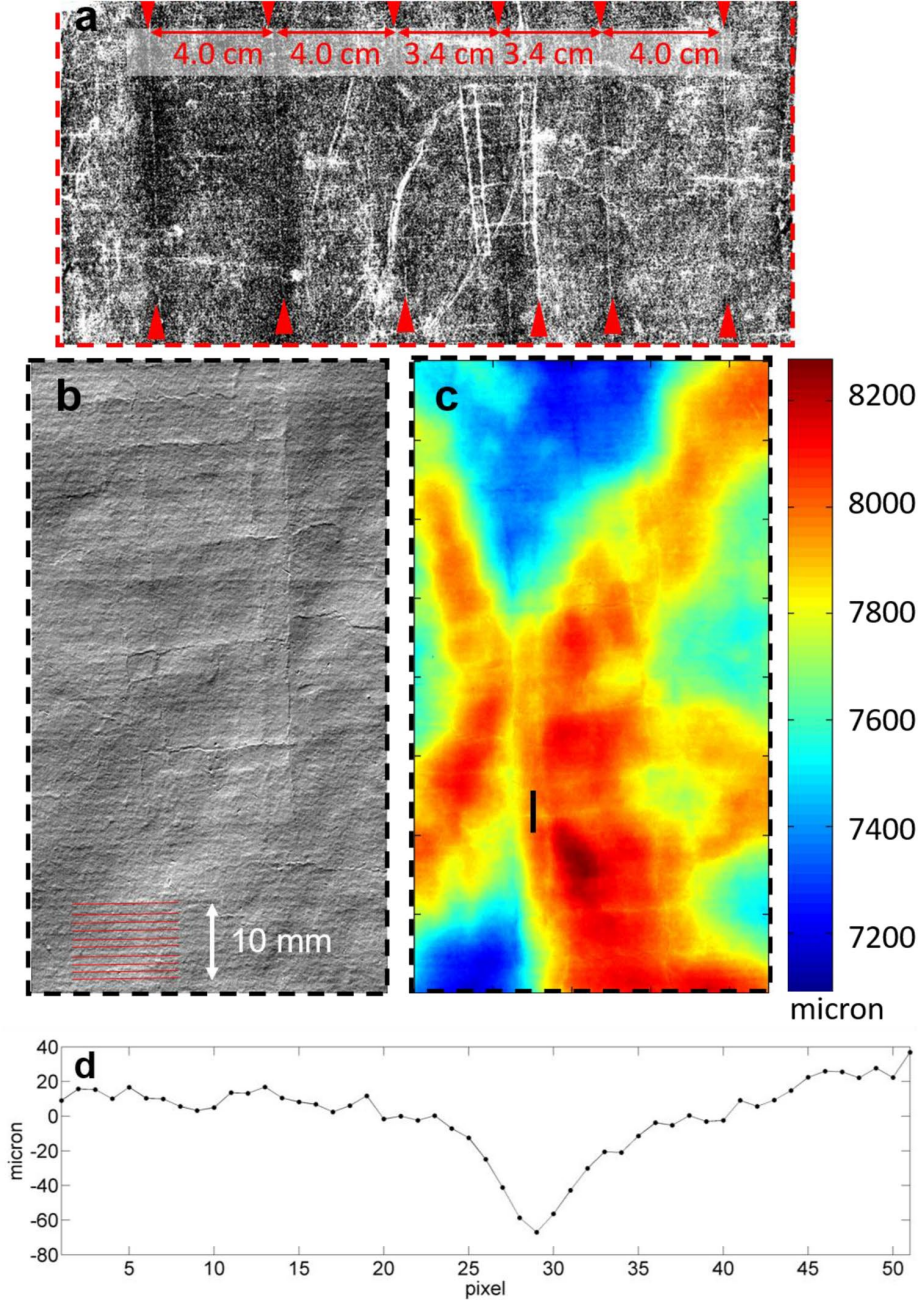
(**a**) Detail (24 × 11 cm) that shows in the inverted 1705 nm NIR image six chain (vertical) lines pointed out by red arrows; (**b**) the raking light image from the topographic map registered with microprofilometry showing 10 laid lines indicated in red; (**c**) area reported in (**b**) in false elevation colours with indicated black vertical segment for quantitative measurement of groove; (**d**) watermark groove profile (after proper shape subtraction) extracted from (**c**).

The Vis–NIR images, the topographic maps obtained from the microprofilometry, and OCT tomocubes allowed us to measure the features of the laid paper used as support. The combined topographic and RIS measurements were profitably exploited to examine the chain lines. Indeed, the detail of the drawing shown as the inverted NIR image at 1705 nm in Fig. 1a highlights the six chain lines pointed out by red dashed arrows. Each chain line at both sides of the watermark is separated by ~ 3.40 cm from the central chain line, while the others are distributed with a step of ~ 4.00 cm. The density of chain lines is consistent with what was previously observed on a drawing by Leonardo^7^.

The watermark on the paper support, in the simulated raking light image in Fig. 1b, shows a ladder motif proving its belonging to the Italian production of paper, specifically from the city of Fabriano, from the beginning of the sixteenth century. Similar watermarks were identified on Raffaello’s preparatory drawing for the *Pietà Baglioni* (Uffizi Gallery) and on a drawing by Michelangelo, presently at the British Museum^30^. Further, the topographic map enables a clearer visualization and measurement of the laid lines (10 horizontal lines are representatively indicated in red in Fig. 1b). Regarding the laid lines’ density, there is a separation of 1.12 mm (± 0.15 mm) between lines. Based on a standard practice for measuring the density of laid lines in historical papers^31^, we count 20.5 lines over 21.43 mm (± 0.82 mm) distance. Both microprofilometry and OCT were exploited to measure other features such as the watermark groove depth. The false colour elevation map shown in Fig. 1c indicates, with black line across the watermark groove, the *x–z* profile (Fig. 1d) used to estimate the groove’ s depth to be ~ 80 μm. Such measure is in line with data extracted by OCT profiling (See Supplementary Fig. S1).

**Figure 2:**
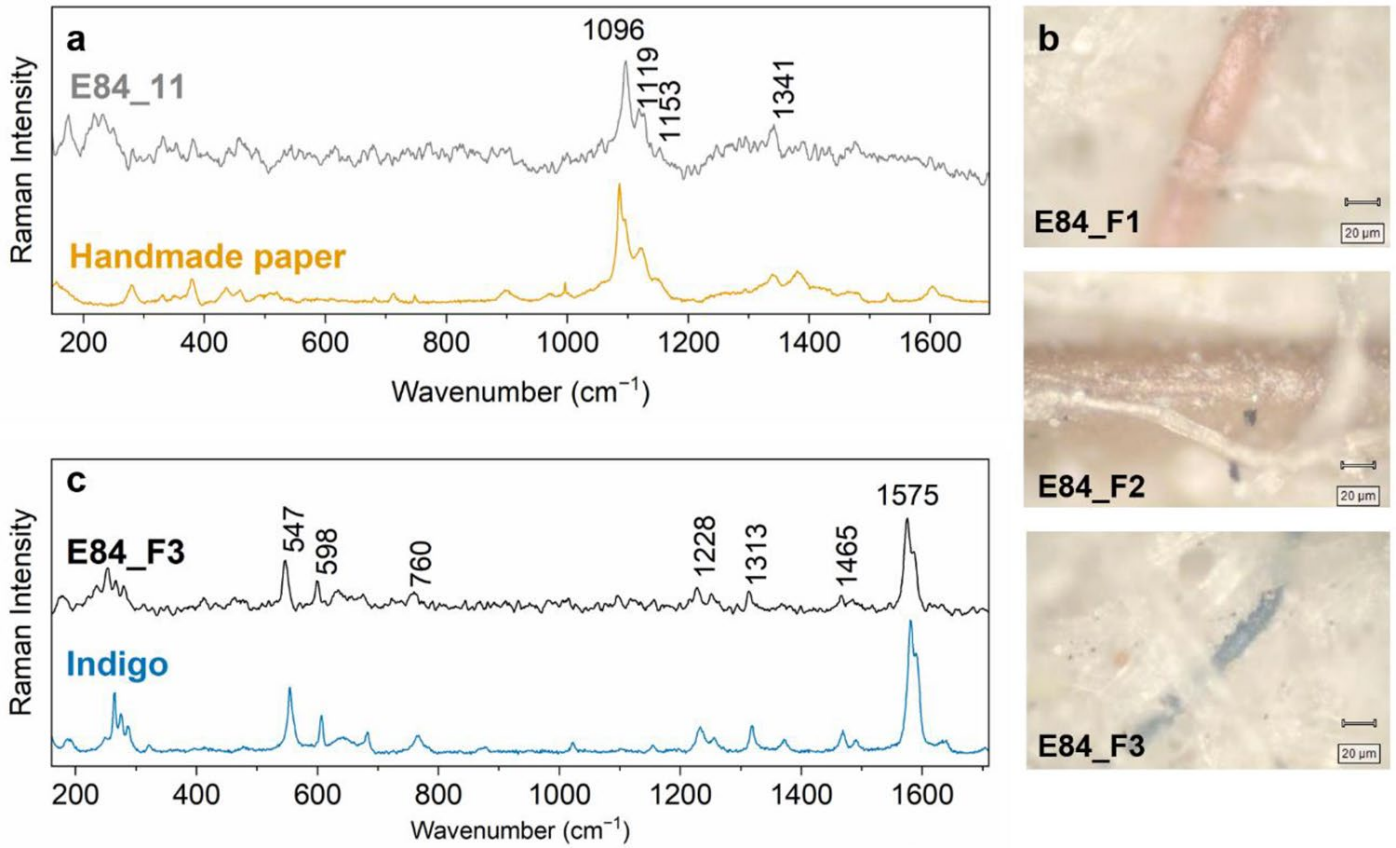
(**a**) μ-Raman spectrum from the paper support (E84_11) compared to the reference of handmade paper, (**b**) microphotographs of the three different fibres, (**c**) μ-Raman spectrum from the blue fibre (E84_F3) compared to indigo reference.

Reflectance spectra in the range 450–2500 nm extracted from different points of the Vis–NIR datacube show the vibrational overtones of the –OH groups form cellulose (see Supplementary Fig. S2). Despite the strong background, the μ-Raman spectra (Fig. 2a) obtained from the paper support show the bands at 1096 cm^−1^, 1119 cm^−1^, 1153 cm^−1^ and 1341 cm^−1^ linked to the cellulose ν-COC-, ν-CC-, δ-CH_2_-, δ-OH groups^32^. The Raman spectra show no evidence of colouring applied to the paper; however, we observed some dyed fibres (Fig. 2b) appearing yellow, orange and blue under the optical microscope. The μ-Raman analysis of the blue fibre (Fig. 2c) indicates the presence of indigo. The other two dyes produced a strong fluorescence upon laser excitation impeding their identification. These coloured fibres were probably part of the old rags exploited as the main material for the paper production^33^.

### Characterization of the drawing media

**Figure 3:**
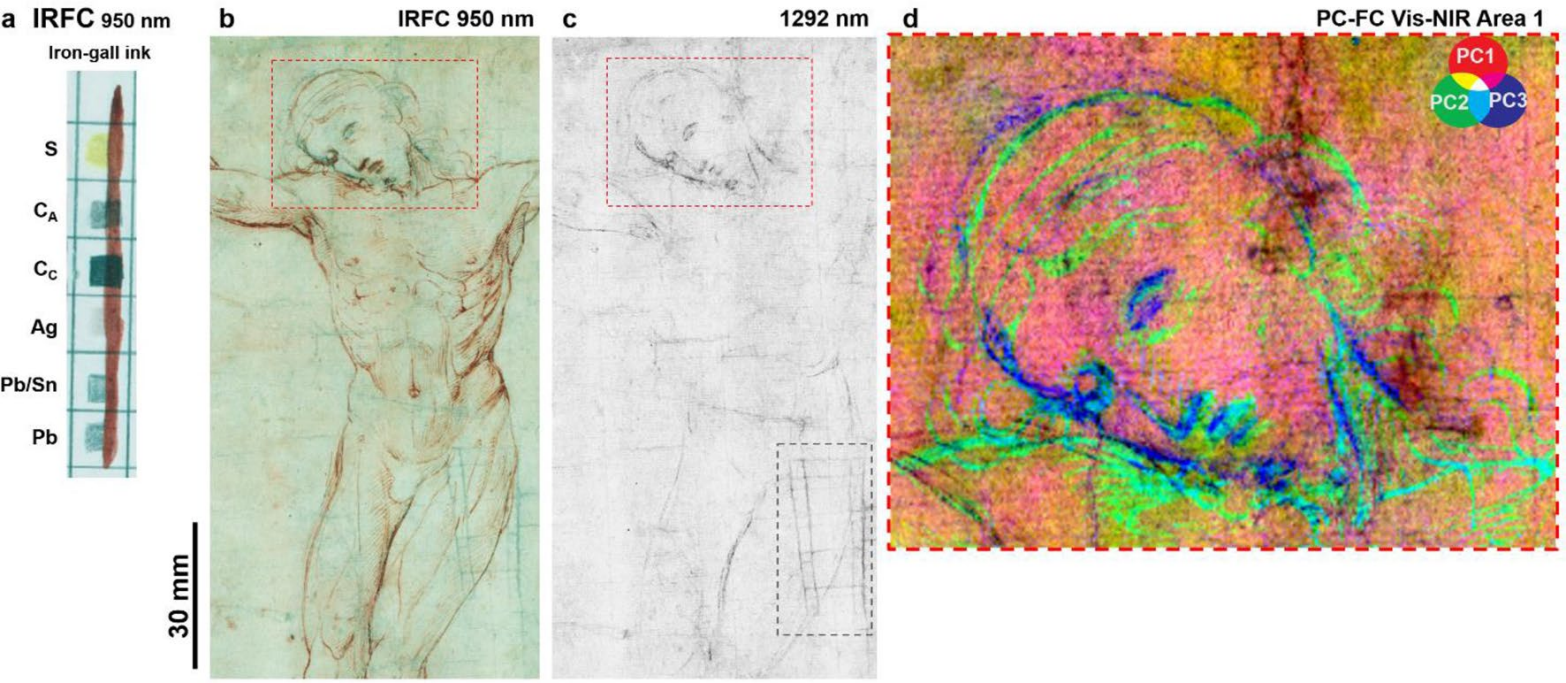
(**a**) IRFC at 950 nm of the reference materials where the iron-gall appears red, while Pb and the carbon-based (C_A_ and C_C_) materials grey/black; (**b**) IRFC at 950 nm where the iron-gall ink lines take on a typical reddish hue; (**c**) NIR image centred at 1292 nm showing the sketch lines realized with a carbon-based material and a lead point and the watermark (dashed rectangle) in bottom right corner; (**d**) PC-FC image (area marked with red dashed rectangle in (**b**) and (**c**) comprising the complete spectral range evidencing the difference between the preliminary sketch (dark blue) and the iron-gall ink drawing (light green).

Data obtained with the reflectance Vis–NIR imaging spectroscopy enabled us to map the variety and distribution of the different drawing media, especially when complemented with Raman spectroscopy and with knowledge gained from previous studies^34^. Indeed, the visual examination under microscope, molecular spectroscopy and the RIS data processing were crucial for the correct classification of the media, since some materials can exhibit similar features in the NIR region (e.g., Ag stylus, Iron-gall ink, see Supplementary Fig. S2). The infrared false colour image (IRFC at 950 nm) in Fig. 3b displays the characteristic reddish colour of the iron-gall ink, as confirmed also by μ-Raman spectroscopy^8,35^. Other line traces that appear bluish-black correspond to the sketch performed by the artists. The reflectograms from the multispectral scanner enabled a detailed examination of the artist’s practice: the images from 1292 nm (spectral region where the iron-gall ink becomes completely transparent, see Supplementary Fig. S2) made the sketch lines evident. The spectral behaviour of the drawing materials suggests different materials for sketching the head and the rest of the figure. The complete transparency of the material used for detailing the head at wavelengths higher than 1292 nm (Fig. 3c)—along with its reddish appearance in IRFC (Fig. 3b), the visual characteristics of the lines under microscope and Raman spectral features—is consistent with iron-gall ink. On the other hand, the persistent visibility of some details of the body in the NIR (Fig. 3c) indicates presence of a more absorbing medium, like a carbon- or metal-based material. The difference between the iron-gall and preliminary sketch media is clearly highlighted in the principal component false colour composites (PC–FC) (Fig. 3d) containing the spectral information over the whole detection range (390–2550 nm). The iron gall appears green while the preliminary sketch blue.

**Figure 4:**
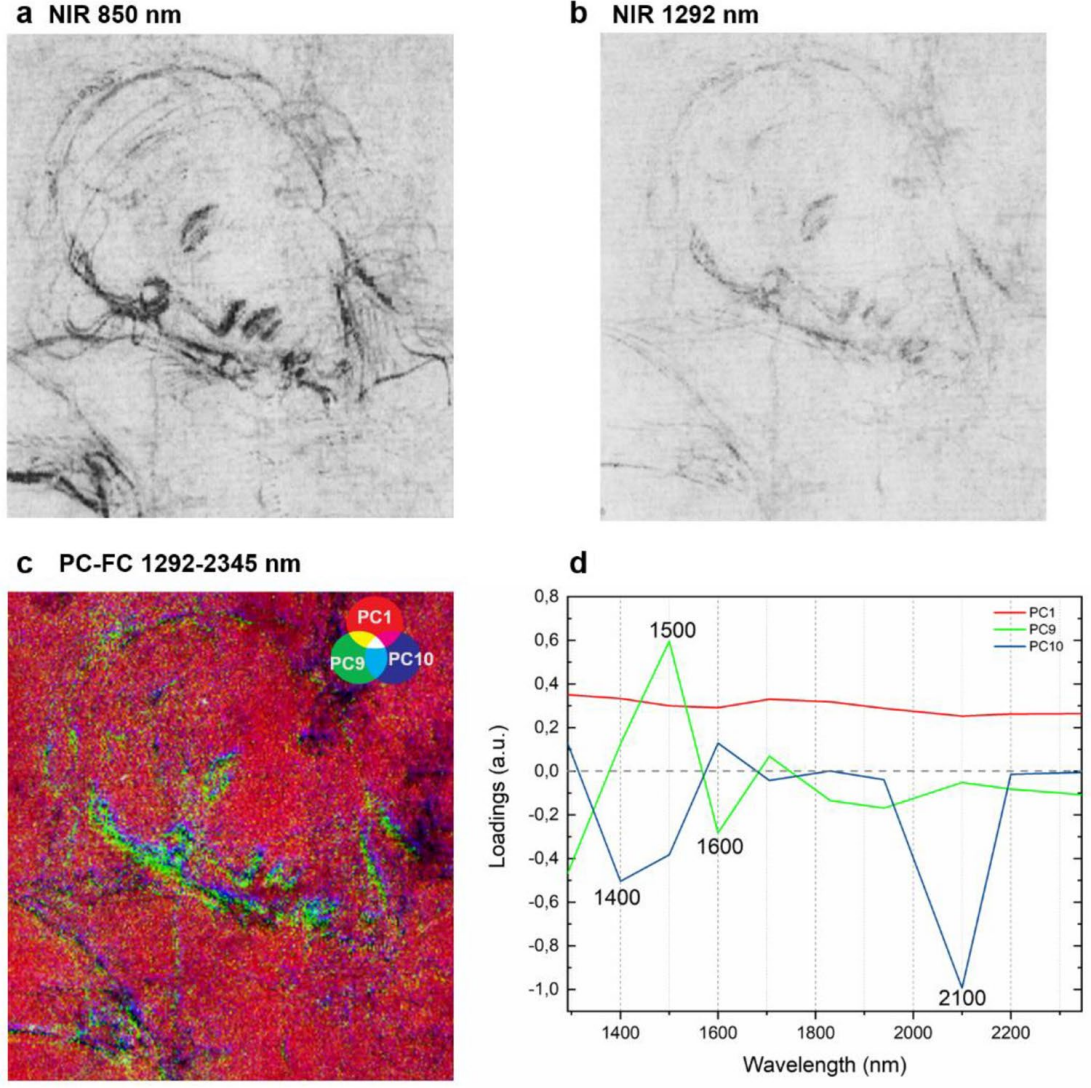
Detail of the Christ’s head: reflectograms centred at (**a**) 850 nm and (**b**) 1292 nm, and (**c**) PC-FC in the range 1292–2345 nm showing the difference between the carbon-based material (light green) and the Pb stylus (dark blue) used for sketching, (**d**) loading plot from PC1, 9, and 10 showing the main characteristic bands for each PC.

A previous examination of the *Cristo Morto* drawing performed with X-ray fluorescence spectroscopy (XRF)^34^ indicated the presence of Pb in correspondence of some areas of the sketch, suggesting the use of a metal point. As observed on the reference materials, it is hard to differentiate between carbon- and lead-based media with NIR multispectral imaging, since they show a similar blueish hue in all the IRFC images (Fig. 3a). Therefore, we performed the PCA on a small area of the drawing (Fig. 4a,b), in the range 1292–2345 nm to eliminate the contribution of the iron-gall ink. The combined PC–FC image (Fig. 4c) allowed us to discriminate between the carbon-based materials, appearing light green, and the lines drawn with the Pb stylus, becoming dark blue. The loadings (Fig. 4d) from the PCA indicate that the spectral regions around 1400 nm and 2100 nm played a particular role in the differentiation of the Pb metal point lines from the carbon in this specific case.

**Figure 5:**
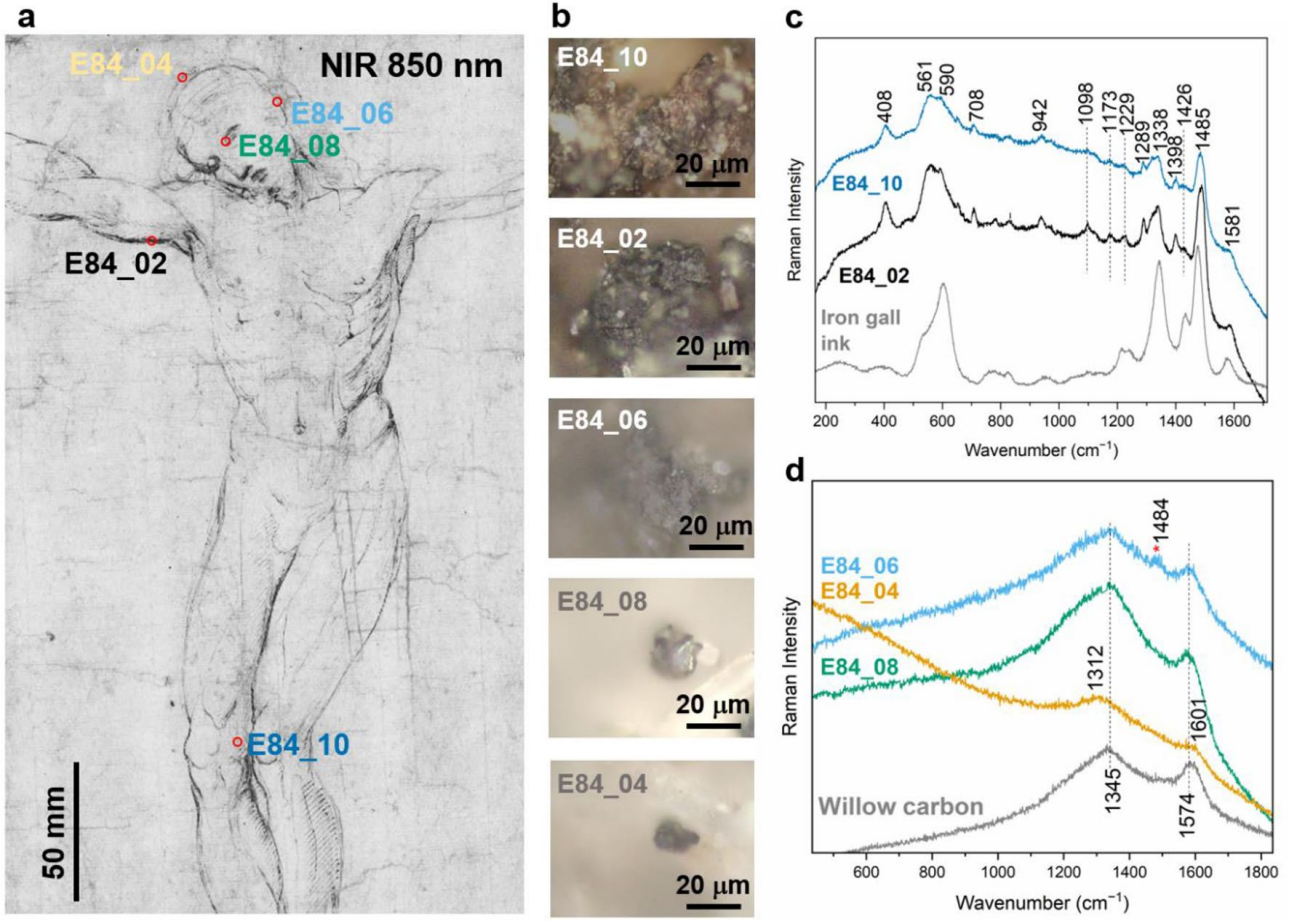
(**a**) Detail of the drawing centered at 850 nm with the measurement points; (**b**) microphotographs of the analysed points; (**c**) μ-Raman spectra of iron-gall ink identified in different points and compared to the reference, (**d**) μ-Raman spectra of identified carbon-based materials compared to the reference (the weak band from iron-gall ink is marked with *).

The μ-Raman analysis (Fig. 5), performed on the lines appearing light green in the PC-FC in Fig. 3d, confirmed the presence of iron-gall ink, while the spectra from the blue lines confirmed the use of carbon-based materials. The spectra in Fig. 5c display the most characteristic bands^36^ at 1485 cm^−1^, 1338 cm^−1^, 590 cm^−1^, 561 cm^−1^ and 408 cm^−1^, related to the presence of iron-gallate (Fe(C_7_O_5_H_3_)·*x*H_2_O) in amorphous form, as suggested by the broad features of the bands^37^. Table S1 reports the assignment of the Raman bands. The spectra in Fig. 5d show the broad bands typical of amorphous carbon with some variability that may indicate different carbon-based materials. In two of the analysed areas (hair and right eye), we identified the bands at 1574 cm^−1^ and 1345 cm^−1^ from the stretching of the CC (so-called G and D band respectively) related to the disorder in the carbon structure^38^. A weak broad band at 1478 cm^−1^ in the spectrum of the hair can be associated to the additional presence of iron-gall ink. Instead, the black line over the head is characterized by the two Raman CC bands shifted respectively to 1601 cm^−1^ and 1312 cm^−1^ (Fig. 5d—E84_04) and previously associated with black chalk^38,39^, a material commonly used by Raffaello for sketching^3,40^.

The results of complementary examination indicate that Raffaello realized the initial sketch with metal- and carbon-based media, and then refined the lines and details with pen and ink. This approach has been previously identified in other drawings by the same artist^3^ and was a common practice for drawings after the sixteenth century^4^. In this drawing, Raffaello used a carbon-based tool to detail the head and the facial elements since it is easier to control the stroke size of the lines^41^. The same material was probably used for two small drawings in the lower-left corner of the verso. Even though it is not possible to establish whether such sketches are contemporary with the principal drawing, this should not be surprising, as it is known that Raffaello used to draw and test the rendering of the drawing materials on the back of the sheets^2,3,42^.

### Conservation history of the drawing

**Figure 6:**
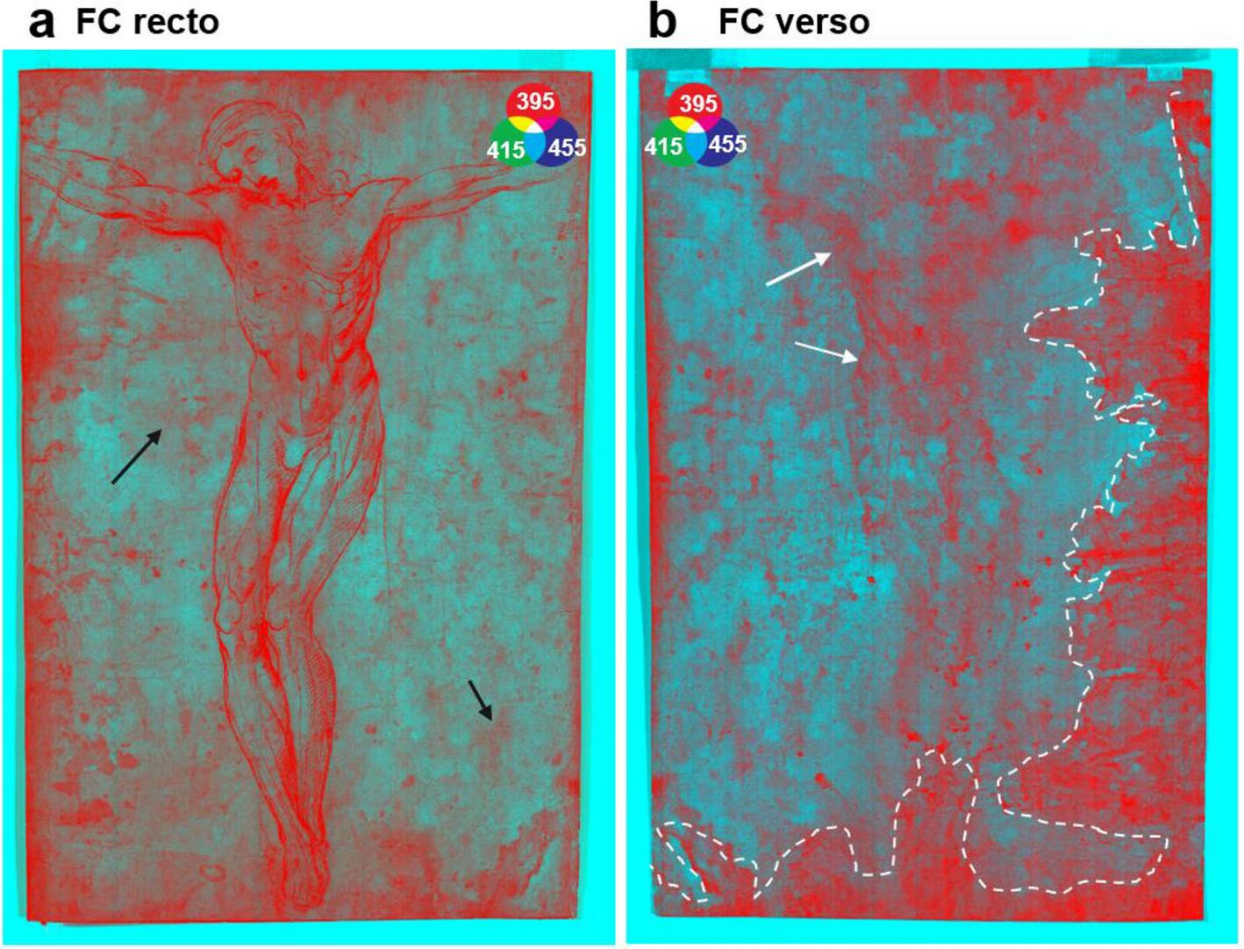
FC composite obtained with the images at 395 nm, 415 nm, and 455 nm, from the Vis–NIR scanner, of (**a**) the recto and (**b**) the verso of the drawing; the dashed areas highlight the zones where the liquid was accidentally spilled. The white arrows marked some areas where the iron-gall ink migrated from the recto to the verso; and the black arrows highlight some details not visible to the naked eye.

The drawing has a complex conservative history, for example paper size modifications and several damages that have occurred over the centuries have been reported^43^. The paper support is affected by extensive deterioration, particularly on the verso, where stains appear due to perhaps accidentally poured unidentified liquid at some point in the past. The FC composite images (Fig. 6a) obtained by merging the images recorded at 395 nm, 415 nm, and 455 nm (where the features of the stains show the highest contrast with the paper support^12^), evidences the damage on the verso. Additionally, the iron-gall ink migration from the recto to the verso not completely visible to the naked eye is highlighted in the FC composite in Fig. 6b.

During the examination of the drawing, some materials related to its conservation history were identified. In the upper-right corner of the paper sheet, there are remains of a reddish material. In that area, the μ-Raman analysis (see Supplementary Fig. S3) revealed traces of cinnabar (α-HgS), which can be related to the presence of a red wax seal^44,45^ or a coloured wax used to fix another paper over the drawing in order to make a copy, as described in Cennini’s treatise^46^.

**Figure 7:**
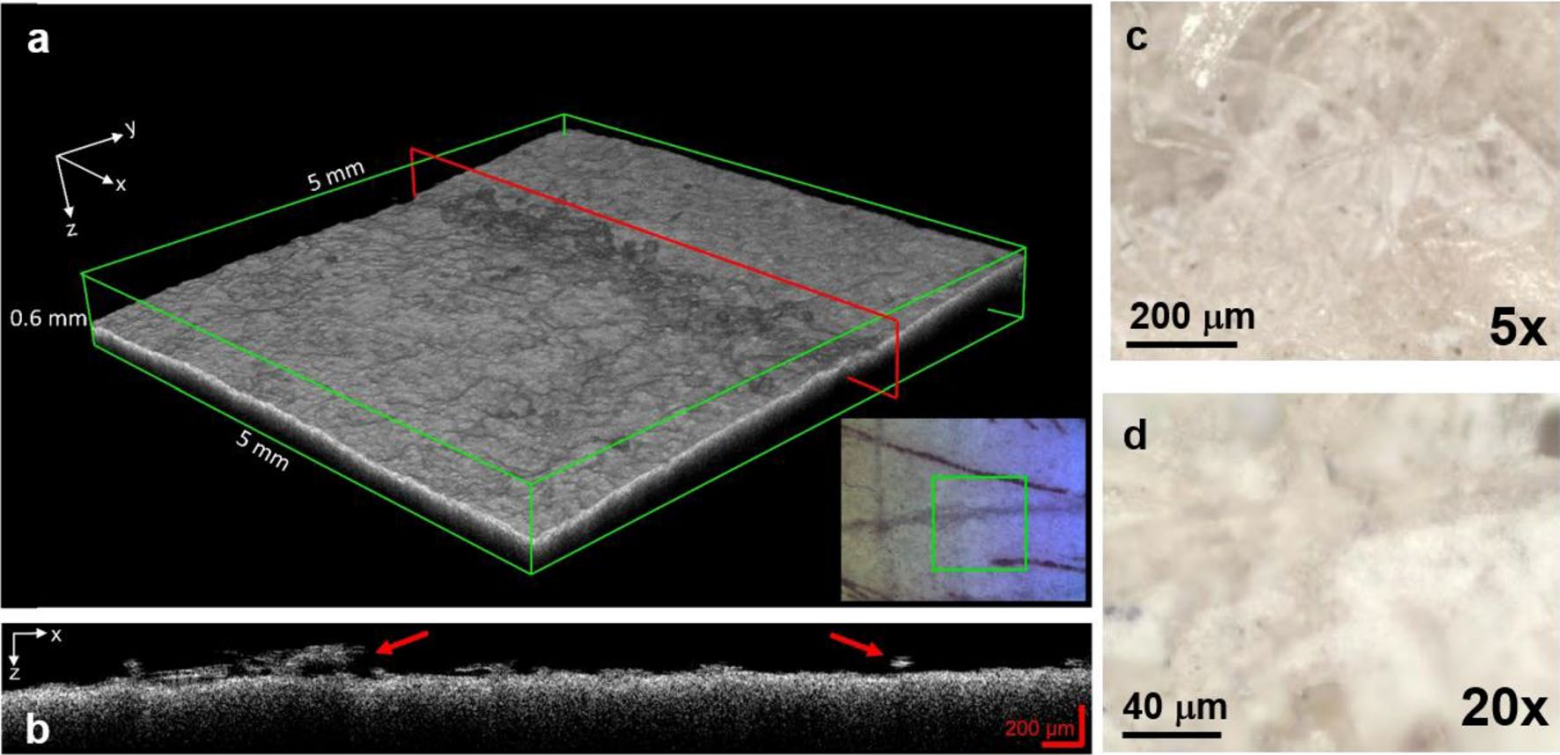
Area of the watermark where the white barium sulphate particles were identified: (**a**) OCT 3D image cube of the groove of the watermark, the red line highlights the y position of the x–z section reported in (**b**), the red arrows indicate the white particle agglomerates. Microphotographs of the particles trapped within the paper fibres in the watermark at (**c**) 5× and (**d**) 20×.

Moreover, within the watermark (Fig. 3c) some white particles trapped between the paper fibres were observed both with optical microscope and OCT (Fig. 7b–d). The agglomerates of a few hundred microns in size were detected in the OCT 3D image cube (5 × 5 × 0.6 mm^3^, voxel size 3.5 mm^3^) reported in Fig. 7a and the x–z OCT section (Fig. 7b) extracted along the watermark groove. The obtained μ-Raman spectrum (sharp peak at 989 cm^−1^ attributable to δ_sym_(SO_4_^2−^) and two broad bands at 616 cm^−1^ and 453 cm^−1^ associated with δ(SO_4_^2−^) group, respectively^47,48^,) can be related to barium sulphate (BaSO_4_) (see Supplementary Fig. S4). Its presence could be related to previous restoration treatments, such as retouching, paper deacidification with barium hydroxide (Ba(OH)_2_) or filler transfer from Japanese paper^49–53^.

## Methods

### The *Cristo Morto* drawing by Raffaello

The drawing entitled *Cristo Morto* (Engl. Dead Christ, BMF Dis. vol. E n. E84 r,v, 360 × 241 mm) by Raffaello Sanzio (1483–1520) dated 1507–1508 is part of the drawings collection from the Marucelliana Library, in Florence, Italy. The *Cristo Morto* was realized in the early years of the Florentine period (1504–1508) of the young Raffaello, when he was particularly interested in the Passion theme and the study of the human anatomy. The drawing, indeed, shows a detailed representation of the body, and has been classified as an *écorché* drawing (i.e., a flayed body) showing the particular interest of the artist in the representation of the superficial appearance of the body influenced by the internal anatomical elements^29^.

### Reference drawing media samples

A set of reference drawing media commonly used by artists in the past was applied on graph paper, both as single layer and in a stratigraphic sequence, to reproduce the artistic process. We used an iron-gall ink (produced by Zecchi following historical recipes), willow carbon (C_A_, by Zecchi), metal points (Pb, Pb/Sn and Ag), sanguine (S) and graphite (C_C_) as references for this study. Additionally, we analysed handmade cotton paper as a reference for the support.

### Reflectance Vis–NIR multispectral imaging spectroscopy (RIS)

The acquisition of multispectral images was performed with an in-house developed scanner that records simultaneously 32 narrow-band images (16 VIS + 16 NIR) in the range 390–2500 nm with a whiskbroom filter-based scanning system^54^. The optical head, composed of a lighting system and catoptric collecting optics, is placed in a 45°/0° illumination/observation geometry, moving with a step of 250 μm and a speed of 500 mm/s. The reflected light is collected by a square-shaped fibre bundle and a set of Si and InGaAs photodiodes, each equipped with an individual interferential filter. The scanner working distance is 12 cm (stand-off distance from the object surface), which ensures safety conditions. A proprietary software controls simultaneously the scanning stages, the autofocus system that maintains the optimal target-lens distance during scanning, and the image acquisition. The monochromatic images obtained are perfectly superimposed, aberration-free, and metrically correct, with a pixel size of 250 μm^55^. The tonal values and brightness/contrast of the inverted NIR reflectogram in Fig. 1a have been adjusted. NIR false-colour (IRFC) images in Fig. 3 in the trichromatic space were obtained by combing the NIR (950 nm), R and G spectral bands (from the colour image^7^) mapped to RGB (R_NIR_G_R_B_G_). Additionally, we performed a multivariate image analysis by means of Principal Component Analysis (PCA) using the whole spectral range recorded (390–2500 nm)^56^. 32 principal components (PCs), explaining the 96.33% of the covariance, were calculated, and the first three PCs, explaining the 87.25%, 5.53% and 2.07% of covariance respectively, were used to obtain false-colour composite images (PC–FC) in Fig. 3 to intuitively display the drawing media differences. Additionally, to highlight the difference between the materials used for sketching, we performed the PCA (10 PC representing the 93% of covariance) in the range 1292–2345 nm. PC 1, 9, and 10, explaining the 62.34%, 0.04%, and 0.01%, respectively, were then combined to produce PC–FC composite image in Fig. 5. False colour composite images were obtained by merging three images centred at 395 nm, 415 nm, 455 nm, respectively, to highlight the damaged areas in Fig. 6.

### Laser scanning microprofilometry

The morphological analysis of the drawing surface was carried out with an in-house laser scanning micro-profilometer. The device is composed of a distance meter (Conoprobe 1000, Optimet, Jerusalem, Israel), equipped with a 50 mm lens, mounted on a XY scanning system. The device allows for a maximum scanning area of 30 × 30 cm^2^ with axial and lateral resolution of nearly 1 μm and 20 μm, respectively, and 8 mm dynamic range. The output is high-resolution topographic map of the measured surface that can be displayed either as a 3D model or an image. In the latter case, image processing based on the application of digital filters and rendering techniques can be performed for enhancing the micrometric details.

### Micro-Raman spectroscopy

Micro-Raman (μ-Raman) spectra were measured directly on the drawing using a Renishaw inVia Raman confocal microscope equipped with a Leica DM2700 optical microscope and a solid-state 785 nm excitation source. After the observation of the sample under the microscope, we focused the laser on the drawing surface and performed the measurements in an extended spectral range of 100–3200 cm^−1^, using a grating 1200 l/mm, and a thermoelectrically cooled CCD detector (spectral range 400–1060 nm) with a spectral resolution of 1 cm^−1^ per CCD pixel (functional resolution of 3 cm^−1^). The laser power on the sample was kept below 1 mW, with typical 10 s integration times and 1–10 accumulations. The data spectra were collected with a 50 × long distance (NA Plan = 0.5; theoretical spot size_785_ = 0.95 μm) and a 100 × long distance (NA Plan = 0.75; theoretical spot size_785_ = 0.63 μm) magnifications, checked for their reproducibility, and processed with Wire5.1 and OriginPro software.

### Spectral-domain optical coherence tomography (Sd-OCT)

We performed cross-sectional analysis of a small area of the watermark using a commercial OCT device (Thorlabs Telesto-II). We used a superluminescent diode with a central wavelength of 1300 nm and a bandwidth about 100 nm. The axial resolution in air is 5.5 μm, while the lateral resolution is 13 μm. The detector consists of a spectrograph made of a diffraction grating and a fast camera. The system is controlled via a 64-bit software preinstalled on a high-performance computer. The 3D scanning path probe with integrated video camera performs high-speed imaging (76 kHz) for rapid volume acquisition and live display. For the OCT 3D image cube reported here, the sample stage provides XY translation and rotation of the sample along with axial travel of the probe. The acquisition field of view (FOV) was 5 × 5 mm^2^, with 0.6 mm imaging depth and a voxel size of 3.5 μm^3^.

## Conclusions

This paper presents methodology based on multi-modal approach to study historical fragile paper-based artefacts, in specific demonstrated on a drawing by Raffaello. We exploited exclusively contactless optical techniques, which is an essential requirement when analysing delicate artworks such as drawings on paper. The techniques allowed us to investigate the original materials, the drawing technique and the drawing’s conservation history.

The reflectance Vis–NIR imaging allowed mapping the drawing media and pre-classifying them according to their features in the NIR region. The PCA analysis of selected areas at specific wavelengths differentiated drawing media, such as Pb stylus and carbon. The false colour composites also contributed to enhance some features of the drawing (e.g., characteristic reddish hue of Iron-gall ink) as well as to document the conservation condition of the support. The morphological analysis with microprofilometry and OCT showed the micro features of the paper support fundamental for the in-depth studies of the Italian Renaissance papers. Despite the fact that neither technique has been so far extensively employed for the investigation of drawings, whose three-dimensionality is not the main characteristics, the obtained analytical results are promising. In fact, laid line density measurements as well as watermarks morphology characterization are crucial data for understanding papermaking process and studying the support.

These results were complemented by μ-Raman spectroscopy, which allowed for the identification of some of the coloured fibres and of the drawing media. Moreover, our and previous XRF analyses revealed that Raffaello employed two dry media to sketch the figure: Pb stylus for the general shape of the body, and carbon mainly for the head and some facial elements. The drawing was completed with iron-gall ink by pen: this procedure has been identified in other drawings by Raffaello and by artists of the same period, including Leonardo.

Our work provides an insight into Raffaello’s *modus operandi* in his production of drawings, specifically during his stay in Florence. The application of both imaging and point-wise analyses proved effective in disclosing in detail the main compositional and technical features of the drawing.

## Supplementary Information

Supplementary Information.

## Data Availability

The data analysed during the current study are available from the corresponding author on reasonable request.
