# Supplementary material for: Non-invasive contactless analysis of an early drawing by Raffaello Sanzio by means of optical methods
Source: Sci Rep. 2022 Sep 16;12:15602. doi: 10.1038/s41598-022-18600-2 (PMC9481574; doi:10.1038/s41598-022-18600-2)
Supplement: Supplementary file 1 — Supplementary Information. [file 41598_2022_18600_MOESM1_ESM.pdf]

Supplementary Information to

Non-invasive contactless analyses of an early drawing by Raffaello Sanzio through optical methods

Diego Quintero Balbas[1], Alice Dal Fovo[1], Letizia Montalbano[2], Raffaella Fontana[1] & Jana Striova[1]

[1]National Research Council—National Institute of Optics (CNR-INO), L.go E. Fermi 6, 50125 Florence, Italy.

[2]Laboratorio di restauro cartacei e membranacei, Opificio delle Pietre Dure-MiC, viale F. Strozzi 1, 50129 Florence, Italy.

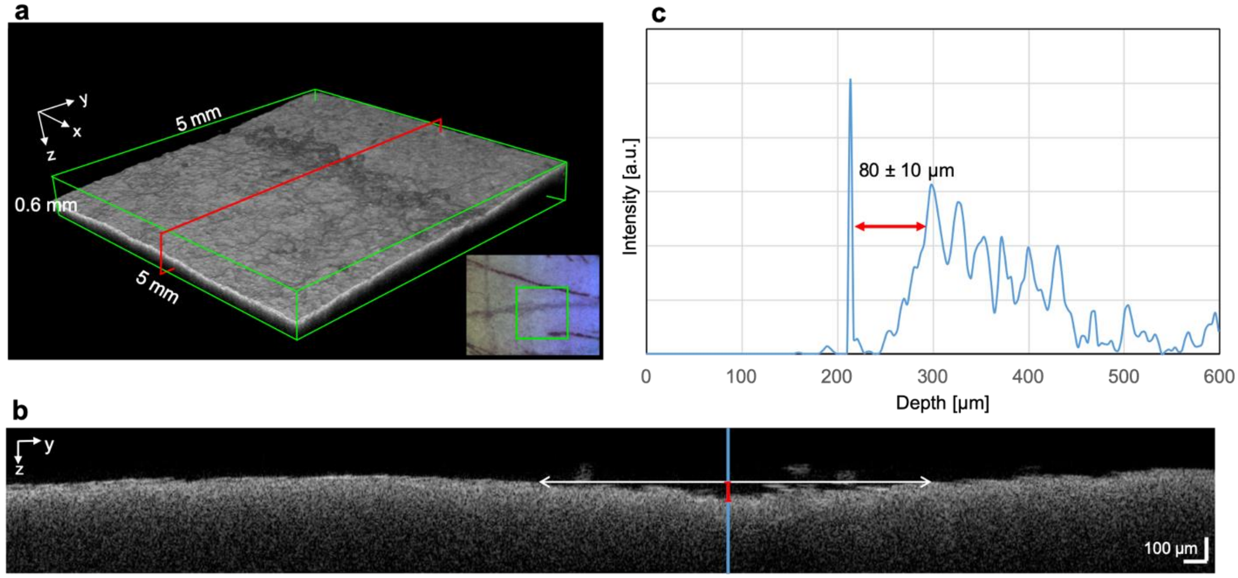

Figure S1. a) OCT tomocube* acquired in the zone of the watermark, the red rectangle indicates the extraction position of b) OCT profile (B-scan) - the vertical blue line indicates the y position of c) depth measurement of the watermark groove (80±10 μm).

The sharp peak at ~200 microns corresponds to the interpolated surface of the paper support.

*replace "tomocube" with: 3D image cube

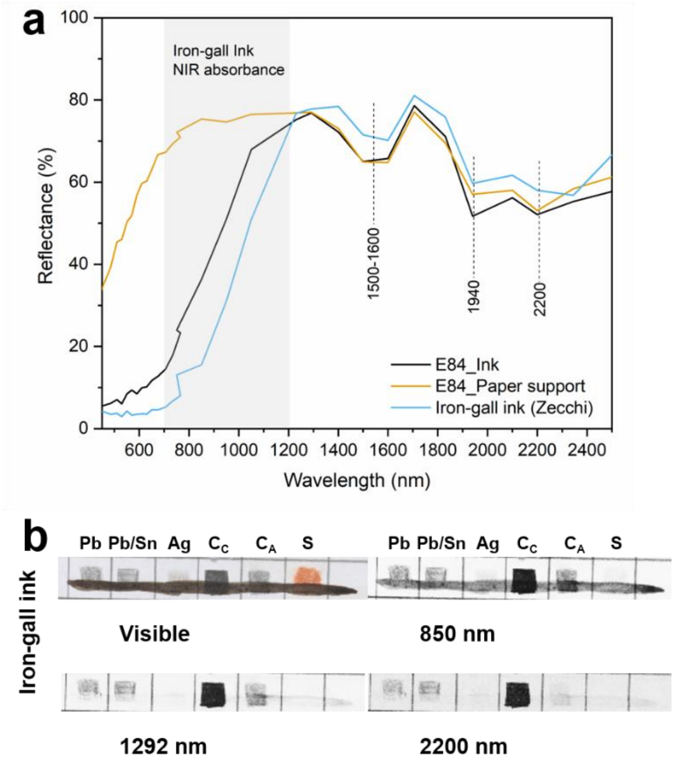

Figure S2. a) Reflectance spectra were extracted from the multispectral datacube over an area with a 10 mm diameter corresponding to about 1250 pixels. b) c) Image of the reference materials in RGB mode and at 850 nm, 1292 nm, and 2200 nm. Pb: lead stylus, Pb/Sn: lead and tin stylus, Ag: Silver metalpoint, C_C: graphite (crystalline carbon), C_A: Willow carbon (amorphous carbon), S: sanguine. A brushstroke of iron-gall ink is applied over the different materials.

Band (cm^{-1})	Assignment [37,38,42–44]
1581	Asymmetric stretching of the coordinated $-COO^-$, Stretching of C=C (ring)
1485	CH_2 scissoring, stretching from C=C from the ring and C-H bending
1426	Symmetric stretching of the coordinated $-COO^-$, bending from O-H and C-H
1398	Stretching of C-O and bending of OH
1338	Stretching of C-O (carboxylic)
1289	Stretching of C=C and C-O
1229	Stretching from C-O (carboxylic)
1173	Stretching of C=C
1098	Aromatic CH mode, stretching C-O (alcoholic)
942	Stretching of C-COOH
708	Ring breathing
590	Iron gallate
561	Out-of-the-plane bending of ring
408	-

Table S1. Raman band assignments from iron-gall ink.

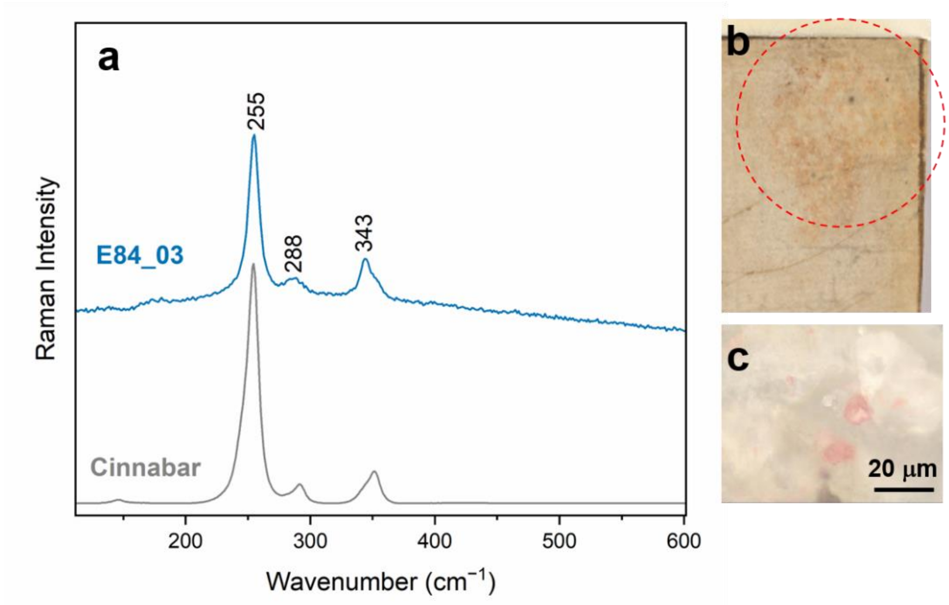

Figure S3. a) Raman spectra of the red particles (microphotograph in inset) with the reference spectra of cinnabar. b) Detail of the RGB image of the recto, the dashed circle indicates the area where the red material is located. c) Microphotograph of the cinnabar particle identified in the surface of the drawing.

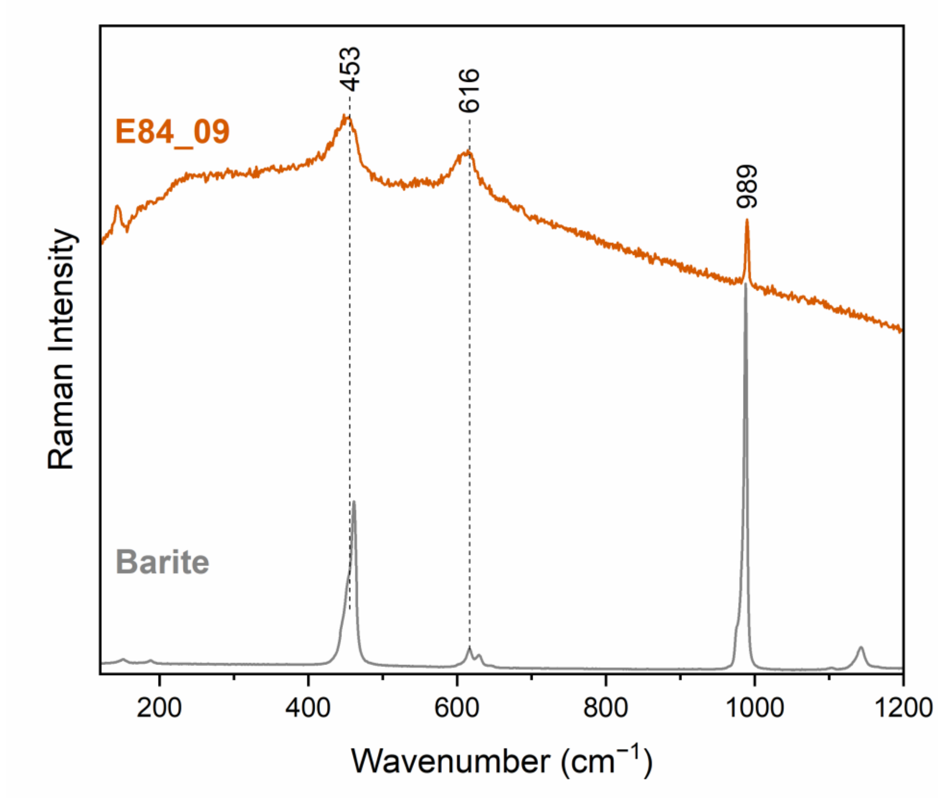

Figure S4. μ-Raman spectrum obtained from the white particles located in the watermark region compared to the spectrum reference of barite (barium sulphate mineral).
